# Supplementary material for: Transport mechanism of DgoT, a bacterial homolog of SLC17 organic anion transporters
Source: EMBO J. 2024 Oct 25;43(24):6740–65. doi: 10.1038/s44318-024-00279-y (PMC11649914; doi:10.1038/s44318-024-00279-y)
Supplement: Supplementary file 1 — Appendix [file 44318_2024_279_MOESM1_ESM.pdf]

Transport mechanism of DgoT, a bacterial homolog of SLC17 organic anion transporters

Natalia Dmitrieva, Samira Gholami, Claudia Alleva, Paolo Carloni, Mercedes Alfonso-Prieto, Christoph Fahlke

Appendix

Table of contents

Appendix Figure S1. 2
Appendix Figure S2. 4
Appendix Figure S3. 5
Appendix Figure S4. 6
Appendix Figure S5. 7
Appendix Figure S6. 8
Appendix Figure S7. 9
Appendix Figure S8. 10
Appendix Figure S9. 11
Appendix Figure S10. 12
Appendix Figure S11. 13
Appendix Figure S12. 14
Appendix Figure S13. 15
Appendix Figure S14. 16
Appendix Figure S15. 17
Appendix Table S1. 18
Appendix Table S2. 19
Appendix Table S3. 21

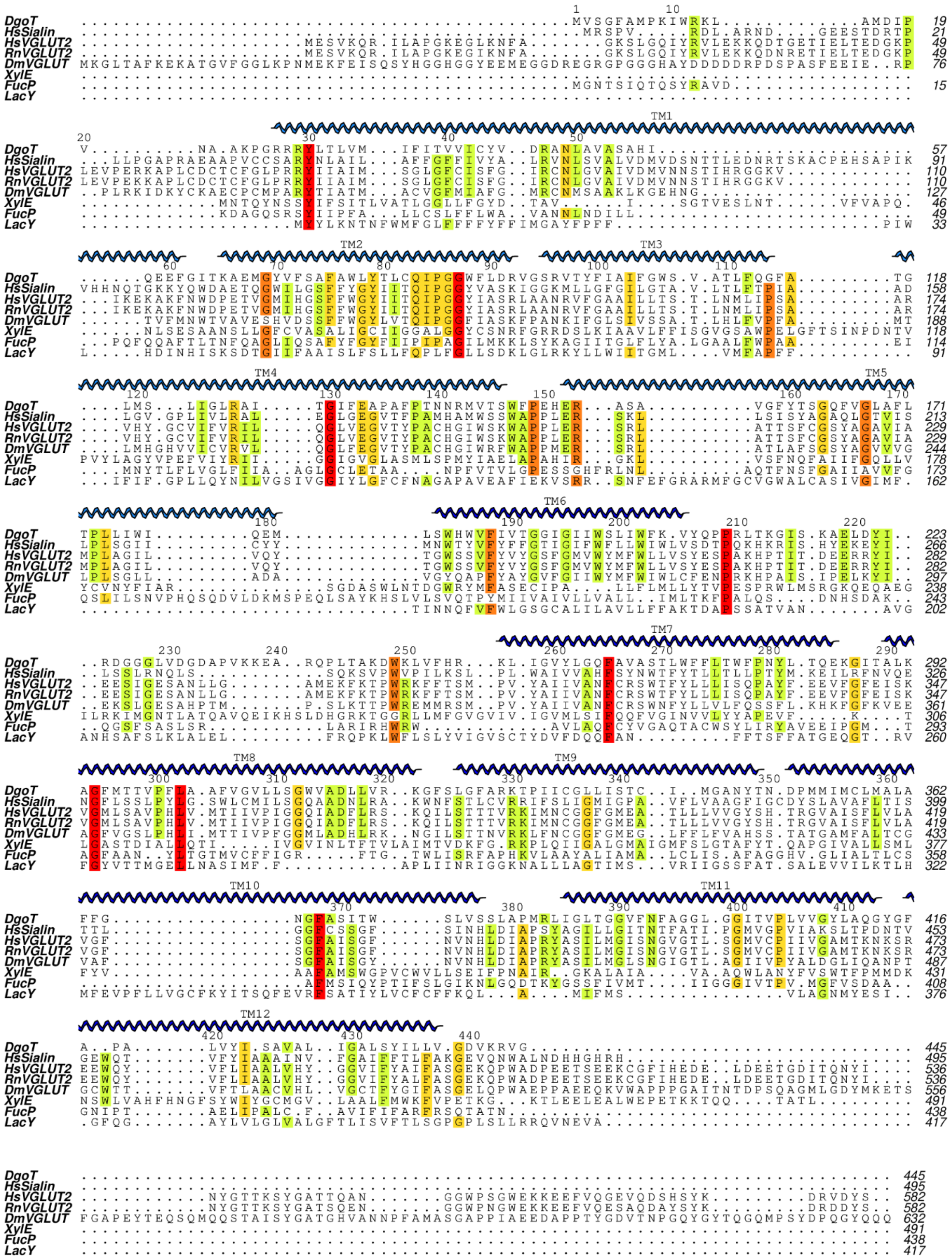

Appendix Figure S1.

Sequence alignment of DgoT and related MFS transporters. Following protein sequences were used for analysis: D-galactonate transporter from *Escherichia coli* (DgoT, AKK15832.2),

sialin from *Homo sapiens* (HsSialin, CAB62540.1), vesicular glutamate transporter 2 from *Homo sapiens* (HsVGLUT2, NP_065079.1), vesicular glutamate transporter 2 from *Rattus norvegicus* (RnVGLUT2, NP_445879.1), vesicular glutamate transporter from Drosophila melanogaster (DmVGLUT2, AAF51256.2), xylose transporter from *Escherichia coli* (XylE, CAD6020582.1), fucose transporter from *Escherichia coli* (FucP, WP_000528603.1), lactose permease from *Escherichia coli* (LacY, WP_000291549.1).

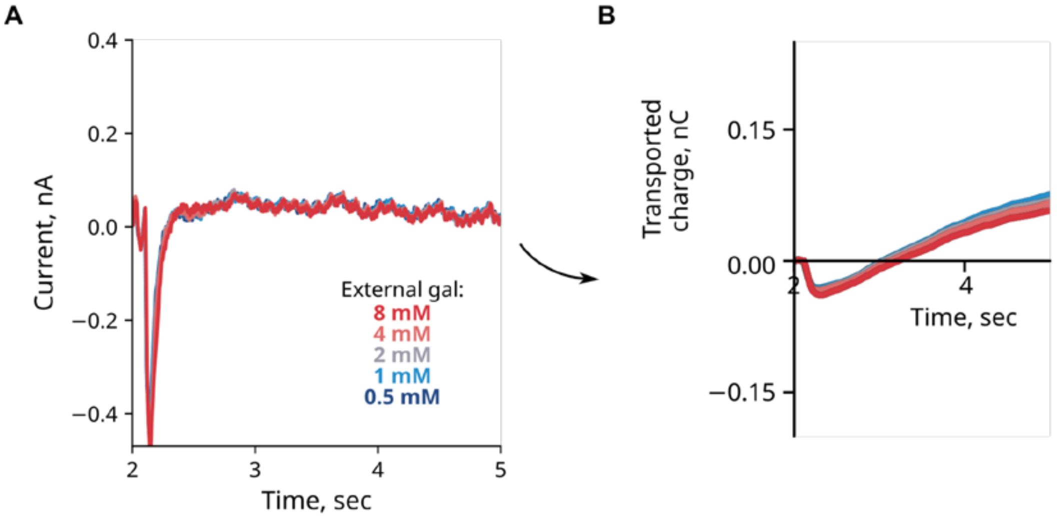

Appendix Figure S2.

Current traces recorded with empty liposomes used as a negative control. A Raw currents recorded with different external solutions. **B** Time dependence of transported charge obtained by integration of current traces.

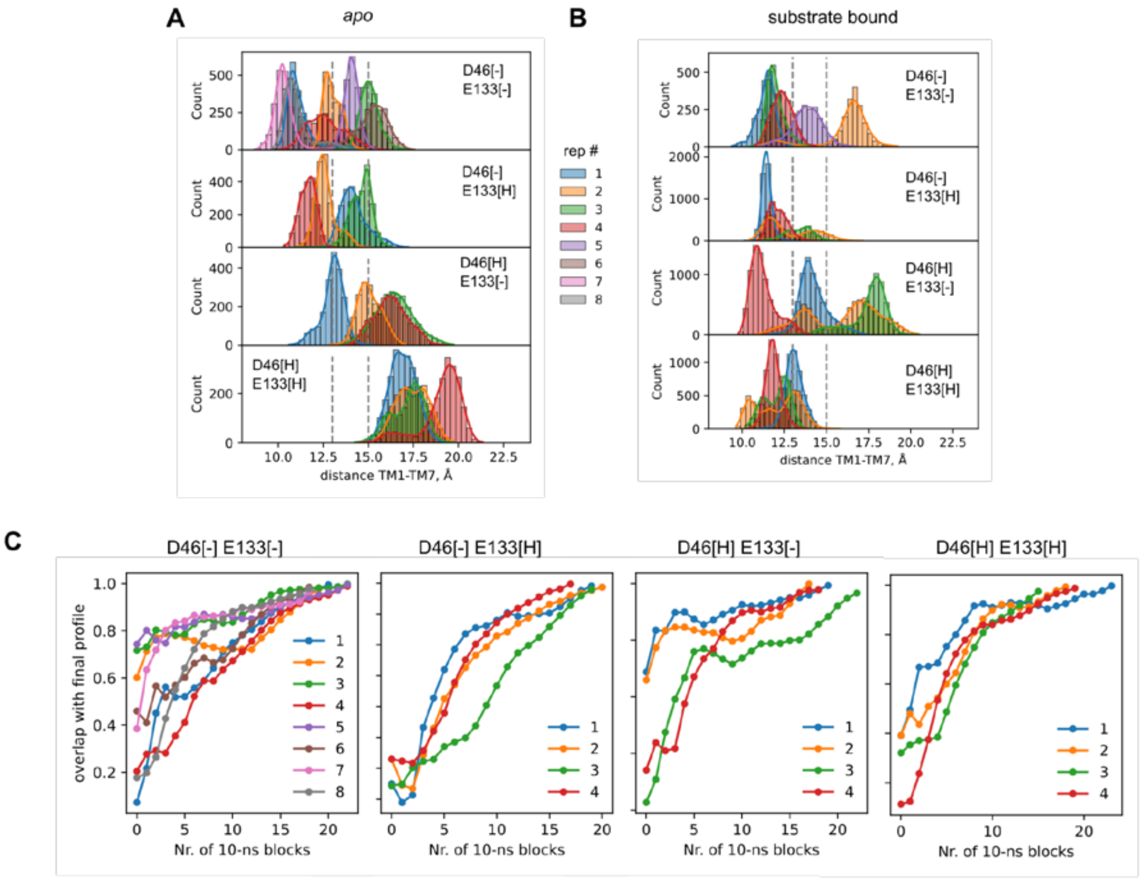

Appendix Figure S3.

Extracellular gate dynamics in unbiased MD simulations with outward-facing DgoT. (A-B). Probability densities for the extracellular gate opening in *apo* (A) and galactonate-bound (B) DgoT with different protonation states of D46 and E133. Distance between TM1 and TM7 was measured as in Fig. 2D and E. (C) Overlap of 10-ns blocks to the final profile plotted against the number of blocks analyzed for *apo* DgoT with different protonation states of D46 and E133

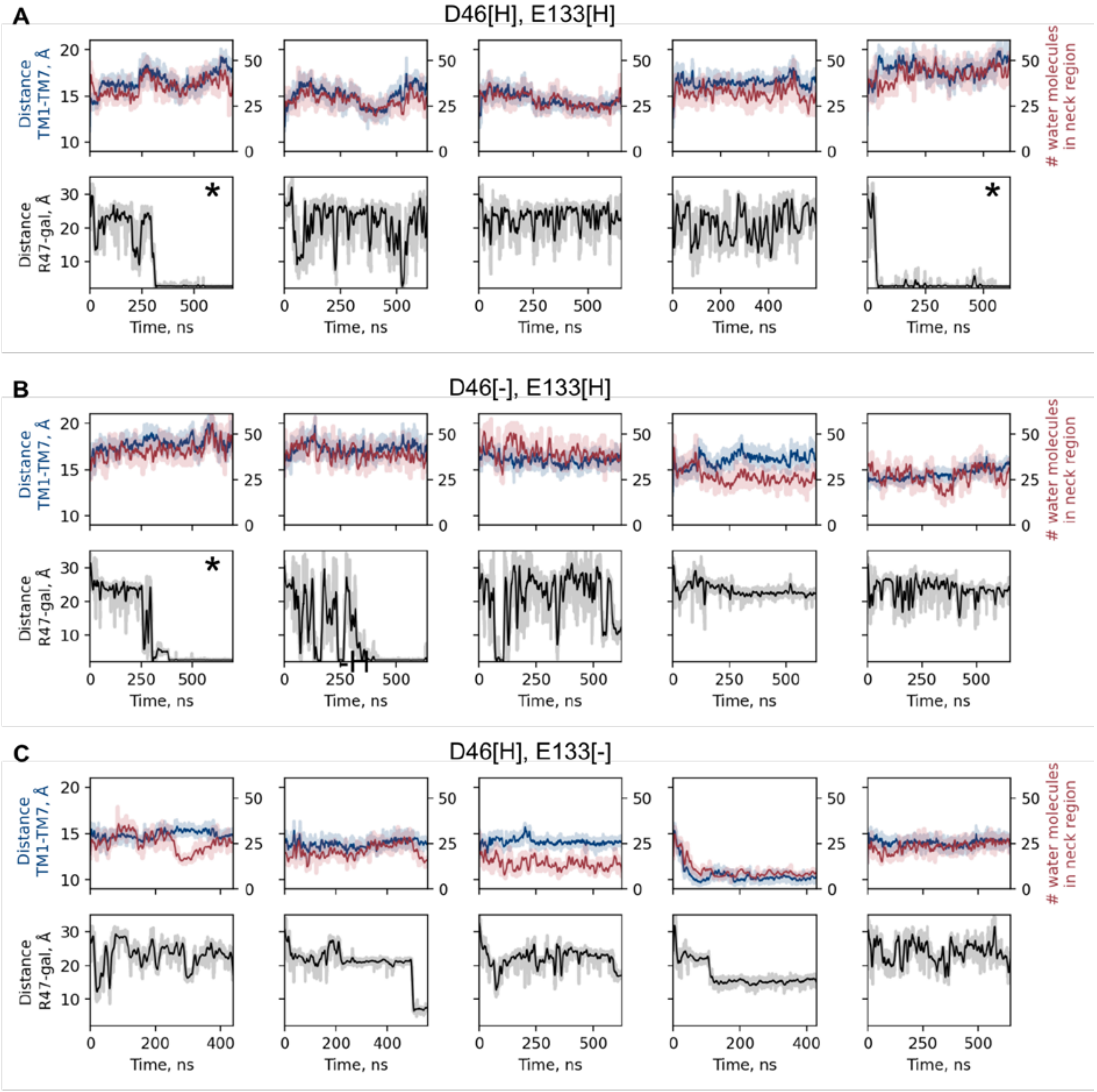

Appendix Figure S4.

Galactonate binding to outward-facing DgoT in unbiased MD simulations.
Time course of extracellular gate opening (measured as TM1-TM7 distance, as in Fig. 2), number of water molecules in 10 Å section near extracellular gate (z coordinate between 58 and 68 Å) and galactonate binding to DgoT (measured as minimum distance between galactonate molecule and guanidinium group of R47) in a trajectories with only E133 protonated (A), only D46 protonated (B) or both D46 and E133 protonated (C). Shaded lines represent raw data from the trajectory, solid lines are moving averages. Asterisks indicate replicas in which binding was observed.

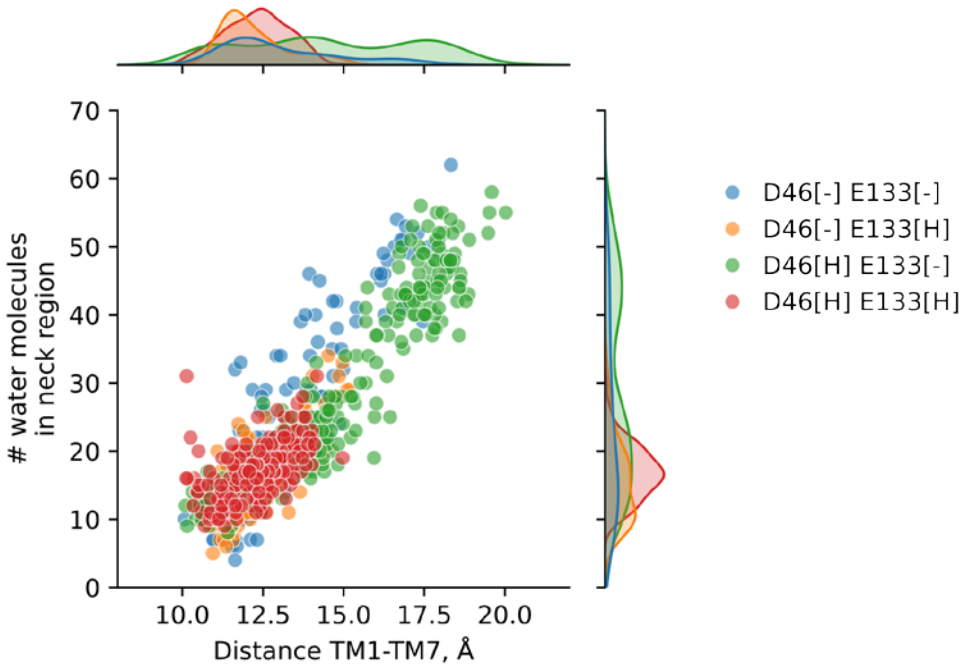

Appendix Figure S5.

Extracellular gate and hydration of the neck region in galactonate-bound MD simulations. Number of water molecules in 10 Å section near extracellular gate (z coordinate between 58 and 68 Å) versus extracellular gate opening in galactonate-bound MD simulations with different protonation states of D46 and E133. The TM1-TM7 distance is measured as distance between the center of mass of Ca atoms of residues 48-52 and residues 271-275.

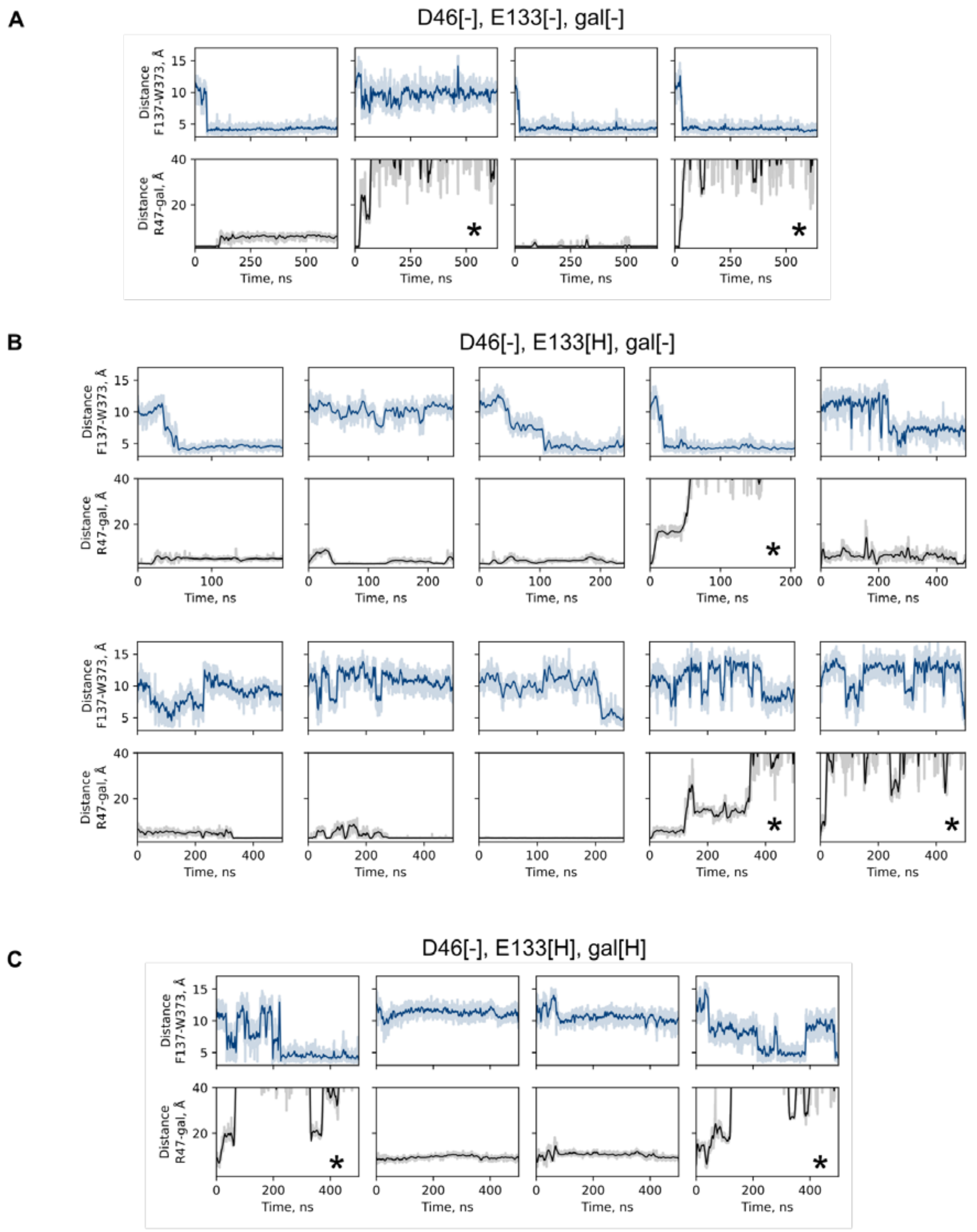

Appendix Figure S6.

Galactonate release from inward-facing DgoT in unbiased simulations.
Time course of intracellular gate opening (measured as F137-W373 distance, as in Fig. 4) and galactonate binding to DgoT (measured as minimum distance between galactonate molecule and guanidinium group of R47) in a trajectories with D46, E133 and galactonate deprotonated (A), only E133 protonated (B) or E133 and galactonate protonated (C). Shaded lines represent raw data from the trajectory, solid lines are moving averages. Asterisks indicate replicas in which release was observed.

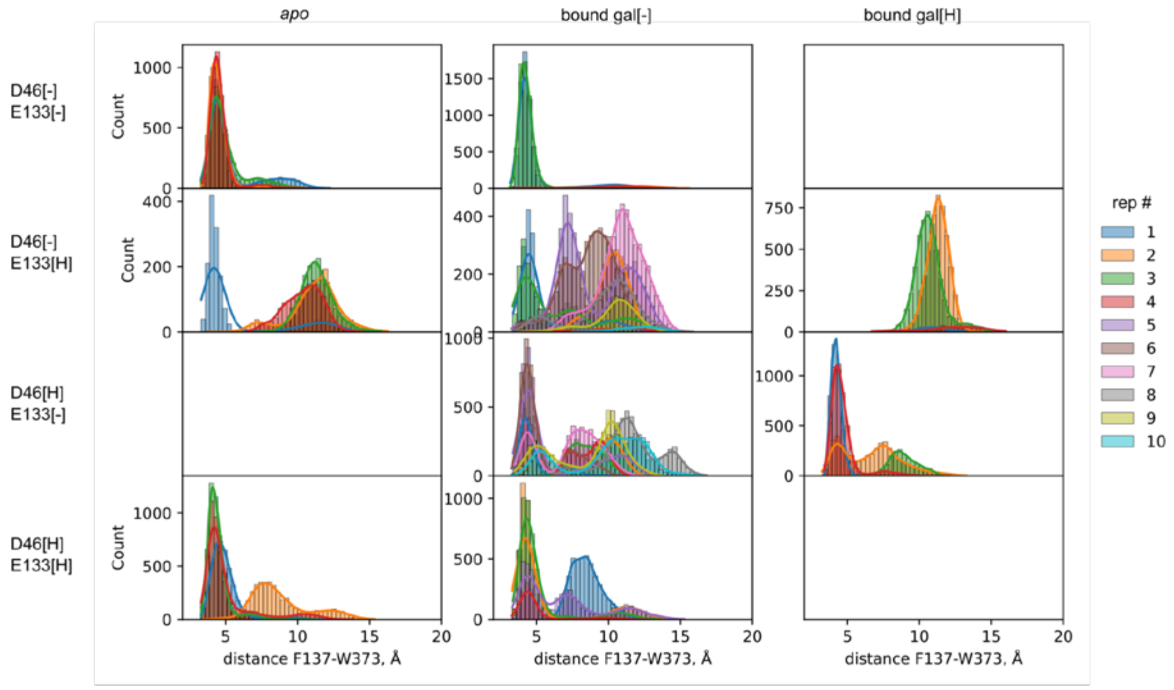

Appendix Figure S7.

Intracellular gate dynamics in unbiased MD simulations with inward-facing DgoT. Probability densities for the intracellular gate opening in *apo* (first column), deprotonated (second column) and protonated (third column) galactonate-bound DgoT with different protonation states of D46 and E133. Distance between F137 and W373 was measured as in Fig. 4D.

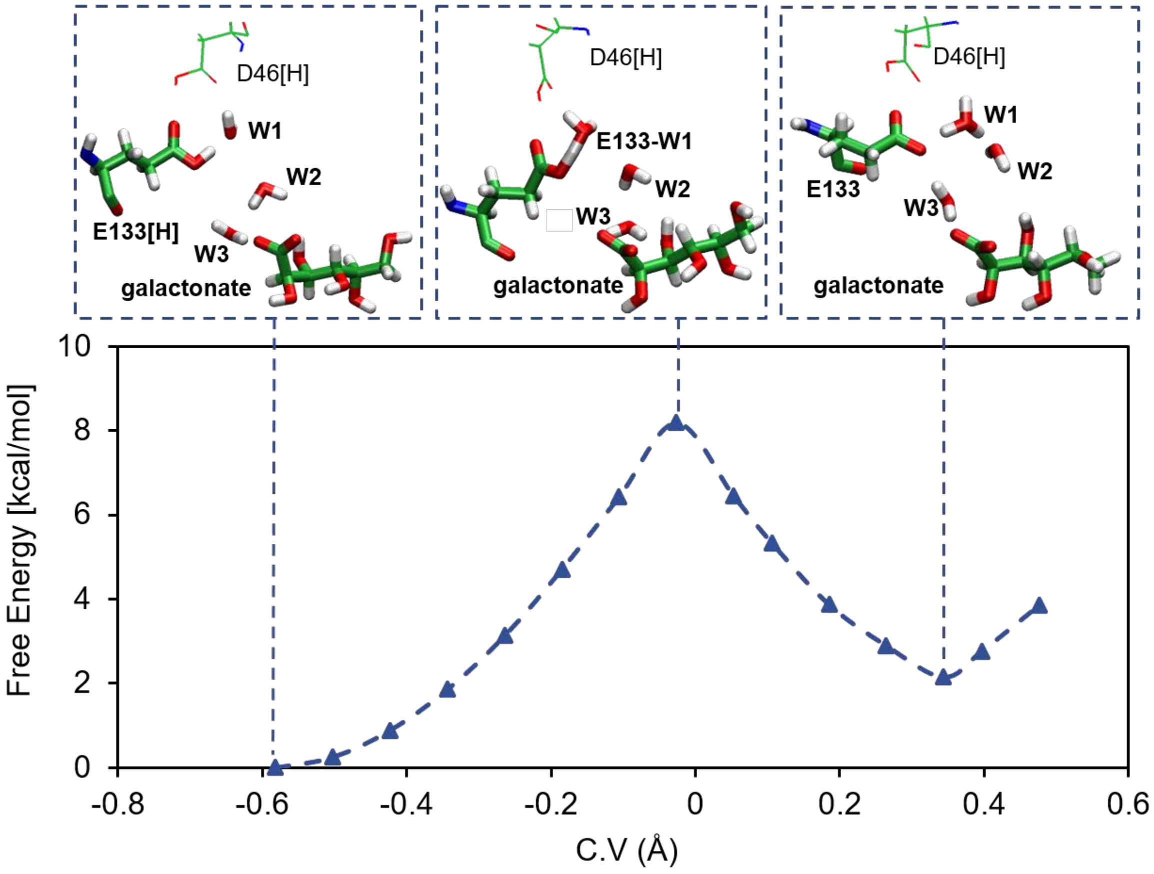

Appendix Figure S8.

Free energy profile for the proton transfer between E133 and the adjacent water molecule (W1) in the *close*-D46 configuration, computed at the QM(BLYP)/MM level. Error bars are omitted since they are smaller than marker size. The insets show representative starting, transition state, and final configurations. QM residues are shown in sticks, while MM residues are shown as lines.

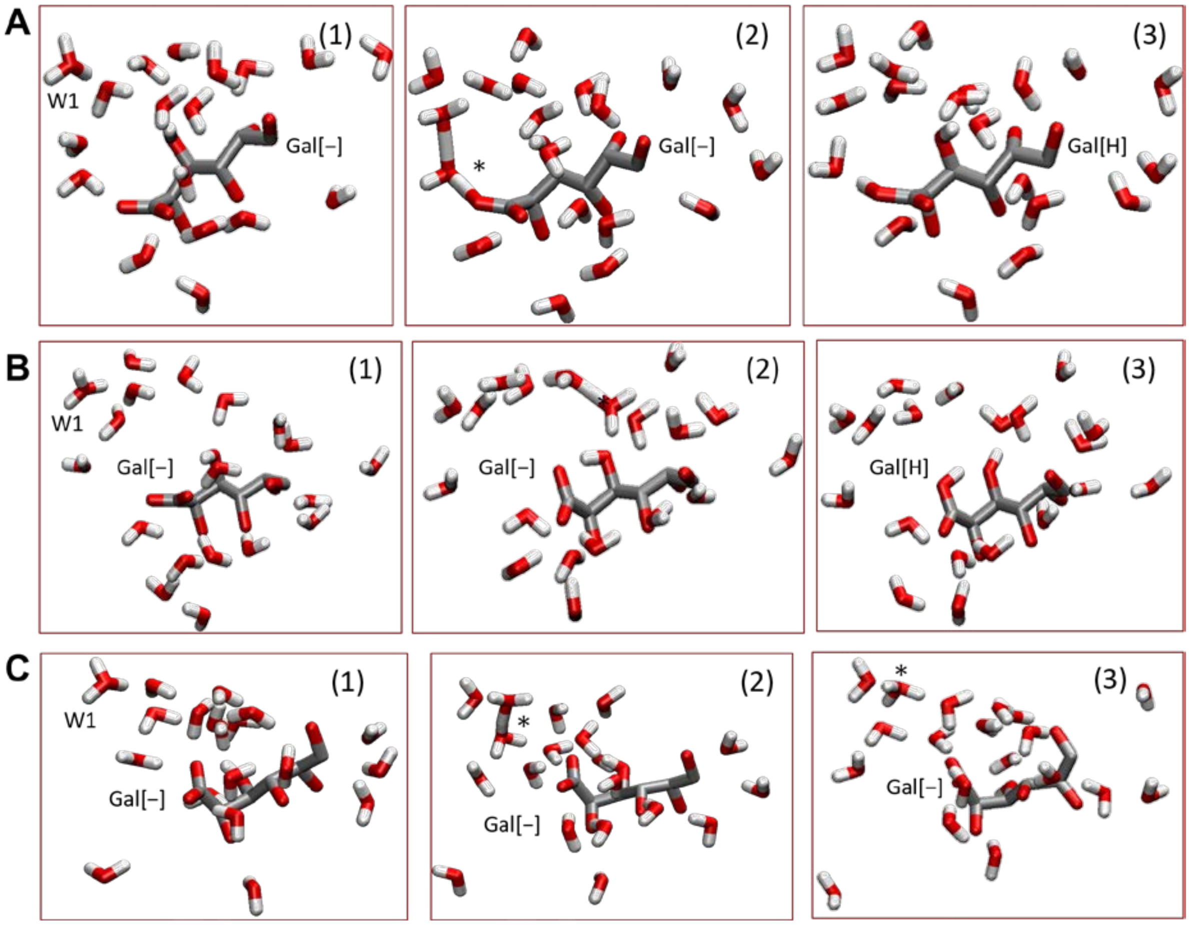

Appendix Figure S9.

Representative structures of the QM/MM MD simulations. A Snapshots of trajectory started from the "snap_4" configuration (see Supplementary Table 2), showing the direct proton transfer from the hydronium ion to galactonate (Gal[H]). Initially the excess proton is located on the first water (W1) and migrates through a 4-water wire until reaching the carboxyl group of galactonate. **B** Snapshots of trajectory started from the “snap_6” configuration, depicting the proton transfer from hydronium ion to galactonate through a 6-water wire and the β-hydroxyl group of galactonate. **C** Snapshots of trajectory started from the “snap_5” configuration, depicting the stablization of the excess protons along the water wire. The asterisk indicates the position of the excess proton in the intermediate structures.

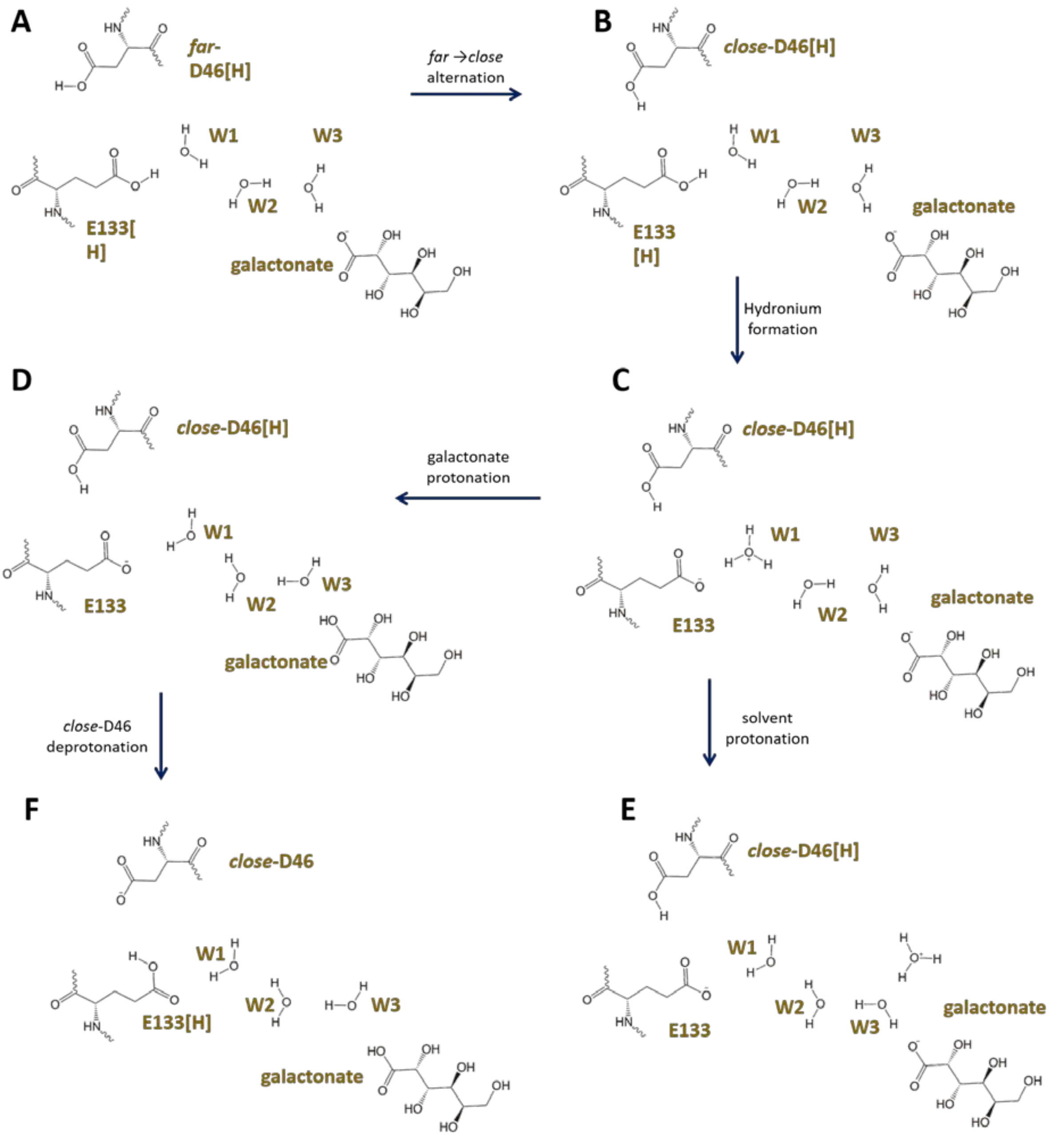

Appendix Figure S10.

Schematic Illustration of the proton release in DgoT. **A** The initial structure featuring protonation of both E133 and D46. **B** Conformational transition of D46[H] from a distant to a close state (*farclose*), leading to the formation of a proton pathway between D46 and E133. **C** Initial proton transfer from E133 to the nearby water molecule (W1), resulting in the formation of a hydronium ion. This excess proton can subsequently be directed either towards the substrate galactonate (**D**) or stabilized within the water network (**E**)**. F** Deprotonation of D46 facilitated by the now deprotonated E133.

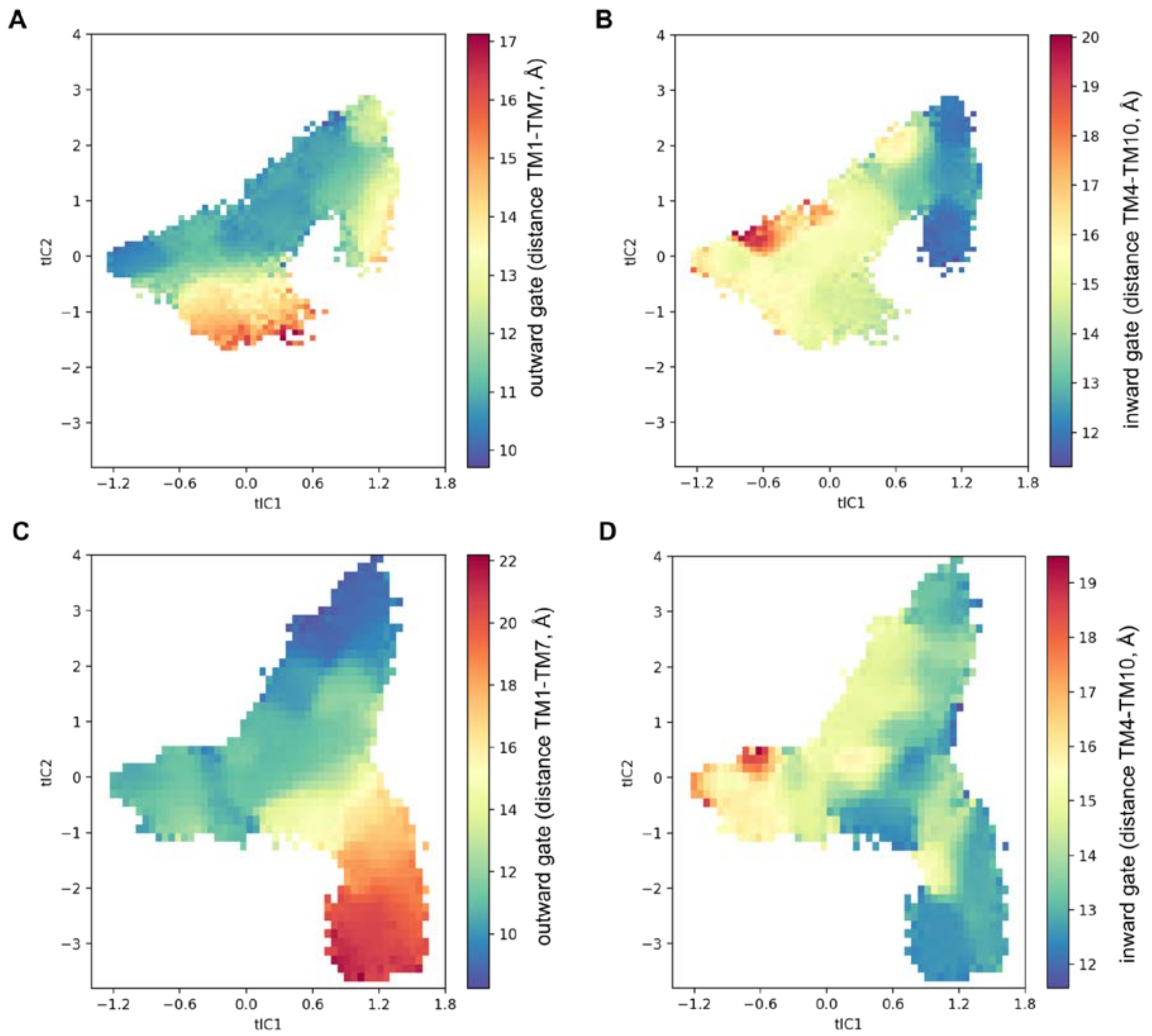

Appendix Figure S11.

Correlation between tICA eigenvectors and distances between gating helices. A and **B** show data for substrate-bound system, **C** and **D** – for *apo* system.

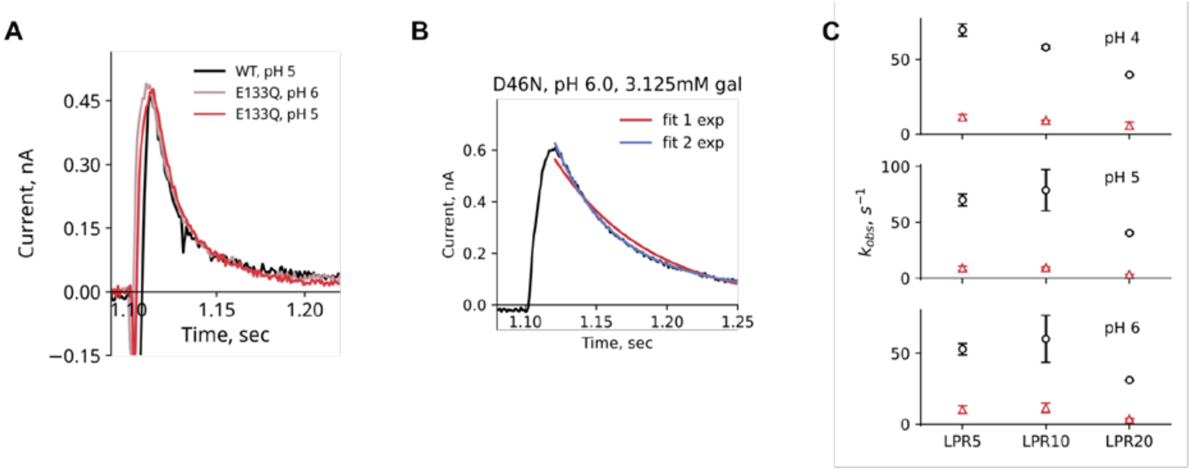

Appendix Figure S12.

Pre-steady-state currents recorded with neutralizing mutants DgoT. a Comparison of representative pre-steady state current recorded with WT and E133Q DgoT at pH 5 or pH 6. **b** Representative D46N DgoT currents with fits to mono- (red line) or biexponential (blue line) functions. **c** Comparison of decay time constants obtained with biexponential fit of currents recorded with D46N DgoT reconstituted in liposomes at different LPR.

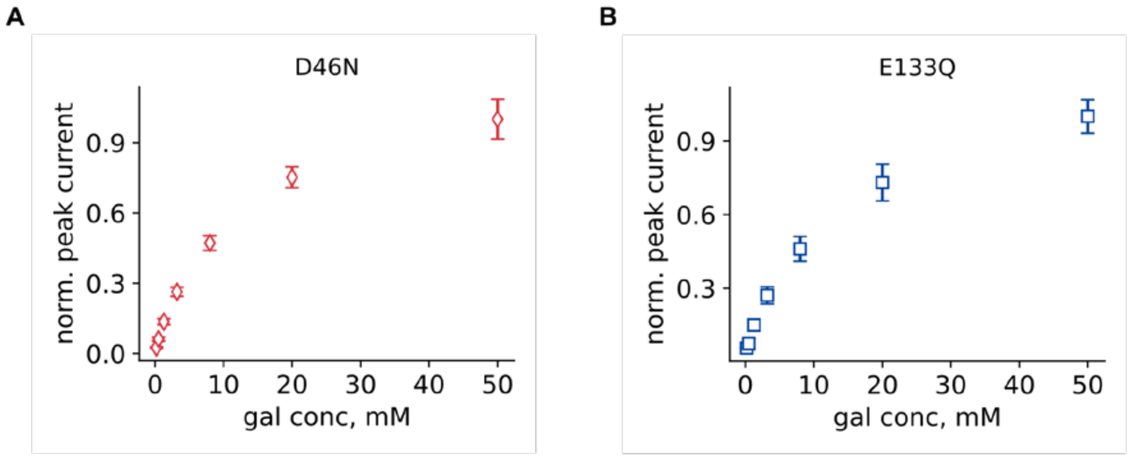

Appendix Figure S13.

Normalized peak currents recorded with D46N (A) and E133Q (B) mutants.

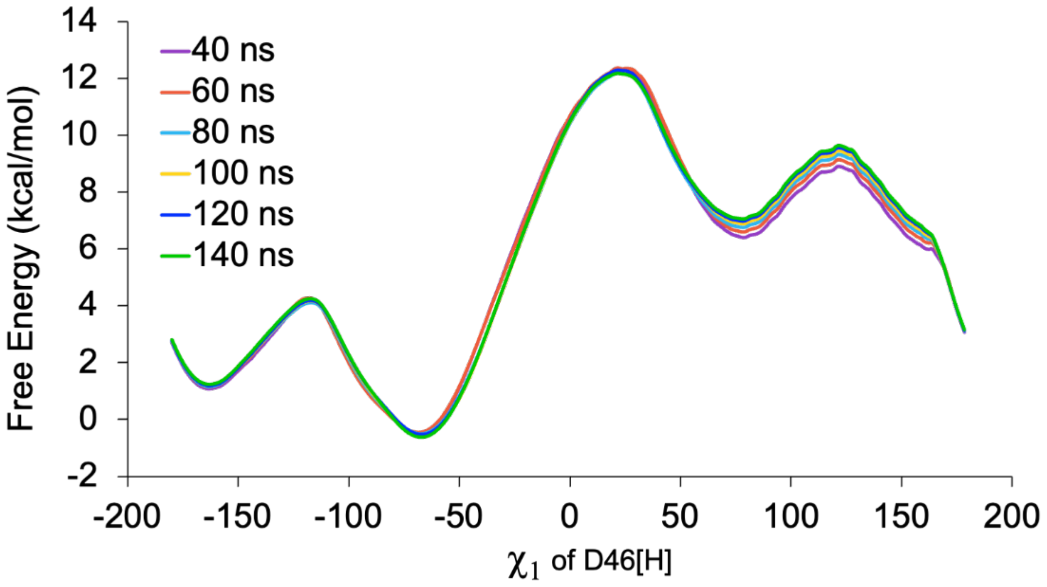

Appendix Figure S14.

Convergence of the free energy profile in classical MTD simulations on the sidechain rotation of protonated D46 as a function of the χ_1 dihedral angle.

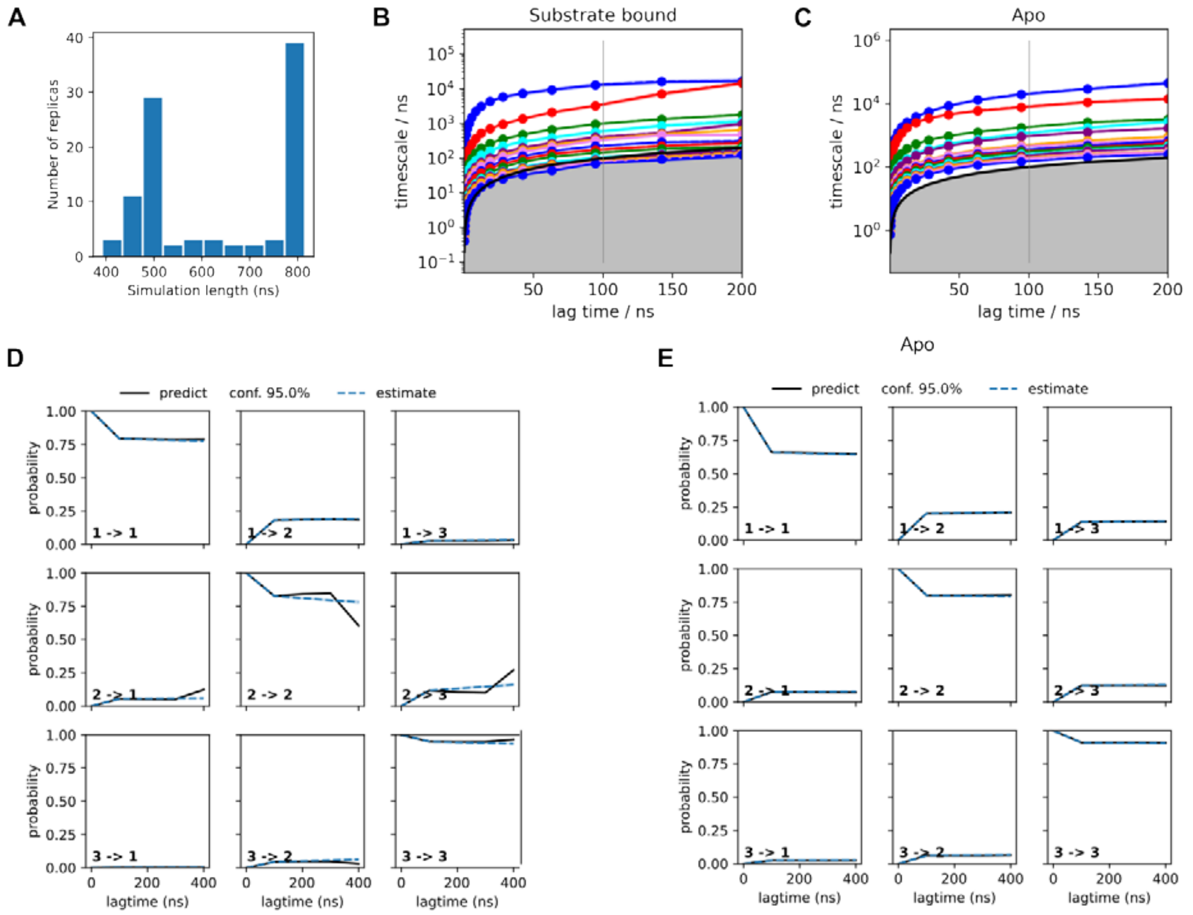

Appendix Figure S15.

Estimation and validation of Markov state modeling of DgoT. A Histogram of lengths of trajectories used for MSM construction. **B, C** Implied timescales plot for systems with substrate bound (b) or *apo* (c) DgoT. Vertical line indicates lag time value chosen for MSM construction. **D, E** Chapman-Kolmogorov test for systems with substrate bound (**D**) and *apo* (**E**) DgoT.

Protonation state: deprotonated (-) or protonated (H)			Total number of replica (500 ns long each)	Number of replica where substrate release was observed
D46	E133	galactonate		
-	-	-	4	2
-	H	-	10	3
H	-	-	10	0
H	H	-	5	0
-	H	H	4	2
H	-	H	4	0

Appendix Table S1.

Substrate release in unbiased MD simulations with inward-facing DgoT with galactonate bound.

Starting configuration	Simulation time (ps)	Proton transfer to galactonate	Water wires	Tau_W1 (ps)	Tau_W2 (ps)	Tau_Wgal (ps)	t_gal (ps)
snap_1	20	yes	4	≤ 0.025	0.125	0.125	0.45
snap_2	30	shared	3	≤ 0.050	0.825	≤ 2.10	4.5
snap_3	35	no	3	≤ 0.025	1.425	0.175	--
snap_4	25	yes	4	≤ 0.050	0.275	≤ 0.025	0.55
snap_5	40	no	3	≤ 0.025	40	--	--
snap_6	35	yes	6	≤ 0.020	0.15	≤ 0.025	14.42
snap_7	35	no	5	≤ 0.020	1.025	15.75	--

Appendix Table S2.

Statistical information pertaining to the proton transfer dynamics simulations for seven representative structures with a hydrated excess proton (snaps 1-7; see Methods). The "Water wire" column denotes the number of water molecules, including the hydronium ion, connecting E133 and galactonate. "Tau_W1" and "Tau_W2" represent the time (in ps) that the proton spends on the initial hydronium ion and its closer neighbor water molecule, respectively, whereas “Tau_W_{Gal}” corresponds to the time that the water molecule adjacent to the carboxyl group of galactonate remains as hydronium ion. "t_gal" (in ps) indicates the total time required for the galactonate to undergo protonation.

**Starting structure**	**Restraint**	**D46 proto-nation**	**E133 proto-nation**	**substrate**	**# replicas**	**Total simula-tion time (μs)**
WT, outward	no	-	-	no	8	3.5
		-	H		4	0.8
		H	-		4	0.8
		H	H		4	0.8
		-	H	100 mM galacto-nate[-] in solution	5	3.2
		H	-		5	2.7
		H	H		5	3.2
		-	-	Bound galacto-nate[-]	5	1.3
		-	H		4	1.5
		H	-		4	2.8
		H	H		4	2
WT, inward	no	-	-	no	4	1.7
		-	H		4	0.7
		H	H		4	1.8
		-	-	Bound galacto-nate[-]	4	2.6
		-	H		10	3.6
		H	-		10	3.9

	χ1 of D46 = 70°	H	-		6	2.6
	no	H	H		5	2
		-	H	Bound galacto-nate[H]	4	2
		H	-		4	1.5
	χ1 of D46 = 70°	H	-		4	1.2
R47Q, outward	no	H	H	no	4	2
				Bound galacto-nate[-]	4	2

Appendix Table S3.

Summary of the classical MD simulations presented in this work.
