# Supplementary material for: Transport mechanism of DgoT, a bacterial homolog of SLC17 organic anion transporters
Source: EMBO J. 2024 Oct 25;43(24):6740–65. doi: 10.1038/s44318-024-00279-y (PMC11649914; doi:10.1038/s44318-024-00279-y)
Supplement: Supplementary file 11 — Expanded View Figures [file 44318_2024_279_MOESM11_ESM.pdf]

Expanded View Figures

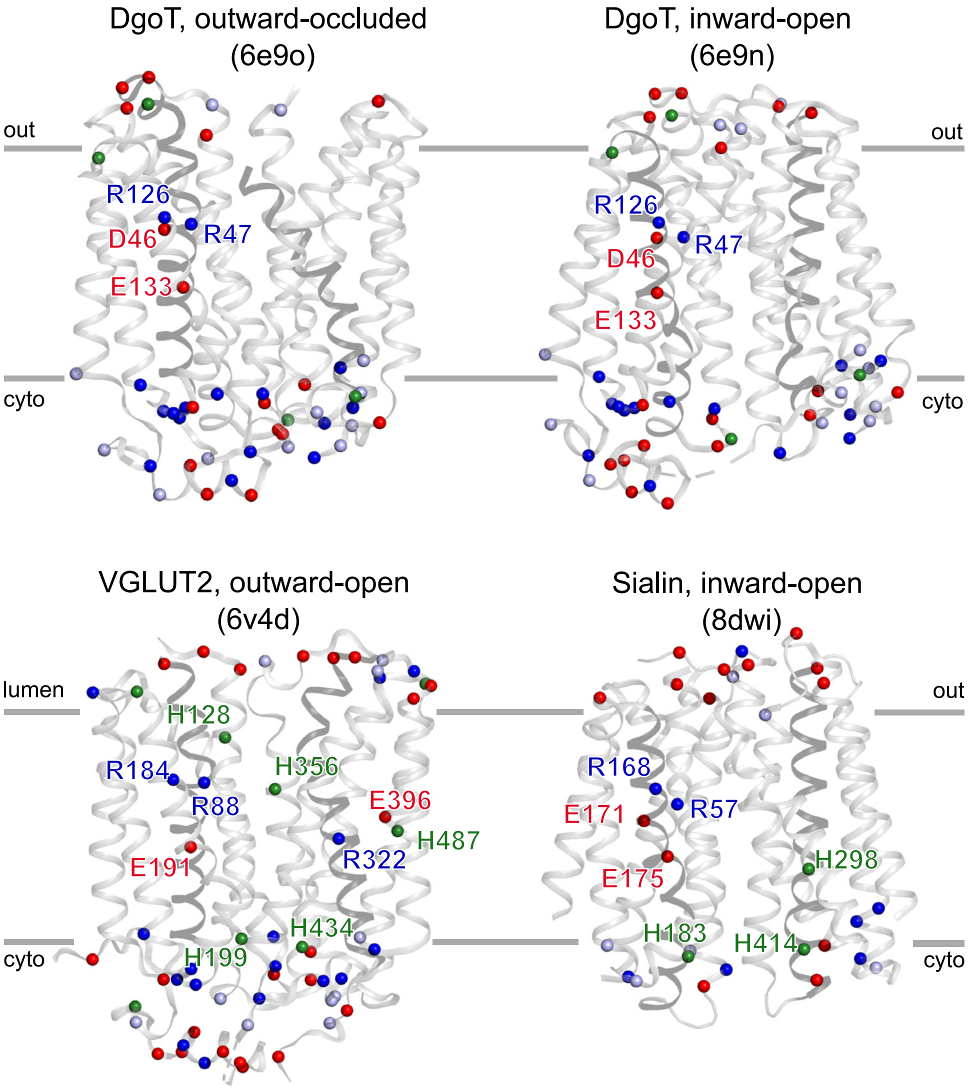

Figure EV1. Structural comparison of SLC17 members DgoT, VGLUT2 and sialin.

PDB codes of the experimentally determined corresponding structures are shown between parentheses. The approximate location of the membrane is indicated with gray lines. Spheres represent the Cα position of Asp and Glu (red), His (green), Arg (dark blue) and Lys (light blue). Charged and titratable residues within the membrane are labeled.

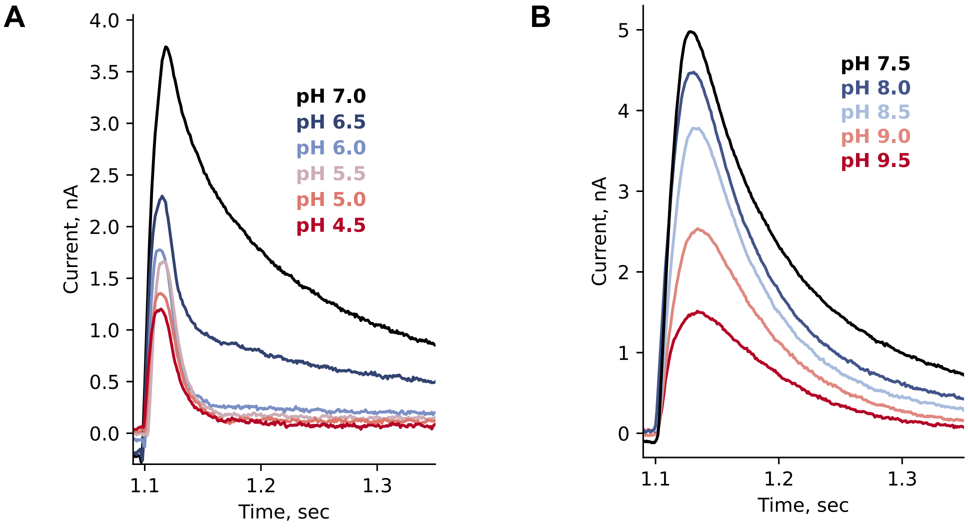

Figure EV2. pH dependence of WT DgoT currents measured by SSME upon application of 10 mM D-galactonate concentration jump.

(**A**, **B**) Representative SSME currents obtained using the low time resolution set up (3 mm sensors) elicited by application of external solutions with various acidic (**A**) or alkaline (**B**) pH values.

 © The Author(s)

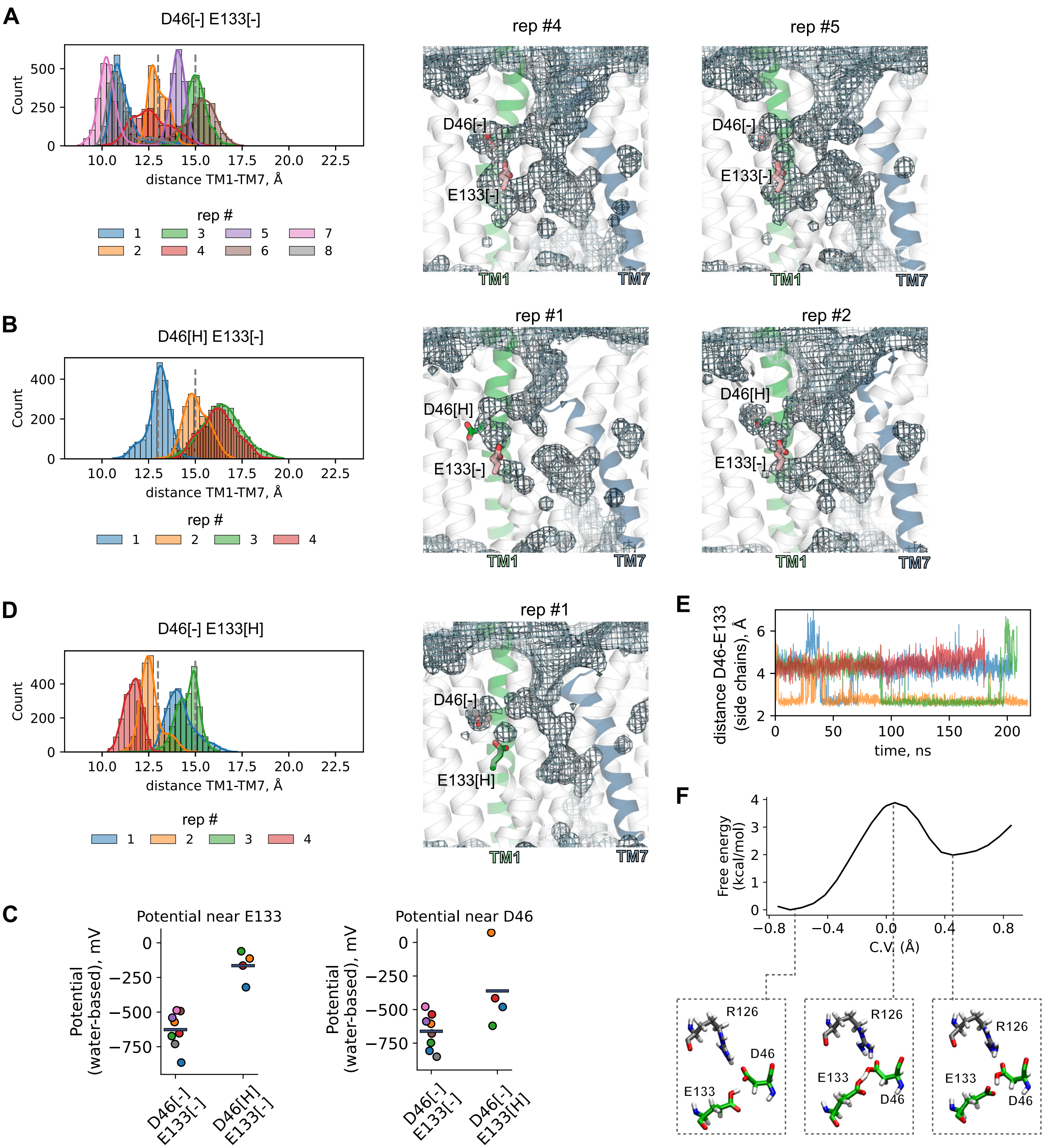

Figure EV3. Protonation of D46 and E133 in outward-facing *apo* DgoT.

(**A–C**) Left: Probability densities for the extracellular gate opening in simulations with *apo* DgoT with deprotonated D46 and E133 ($n = 8$) (**A**), protonated D46 and deprotonated E133, $n = 4$ (**B**) or deprotonated D46 and protonated E133 ($n = 4$) (**C**). *Right*: Water occupancy map in selected replicas, contoured at an occupancy level of 0.2. (**D**) Time course of minimum distance between carboxyl groups of D46 and E133 in simulations with *apo* DgoT with deprotonated D46 and protonated E133. (**E**) Free energy profile for the proton transfer between D46 and E133, computed at the QM (BLYP)/MM level. The insets show representative starting, transition state, and final configurations. Error bars are omitted since they are smaller than the marker size. (**F**) Electrostatic potential near the carboxyl groups of deprotonated D46 (left) and E133 (right) in the unbiased MD simulations. Each data point is an average potential from a single trajectory.

© The Author(s)

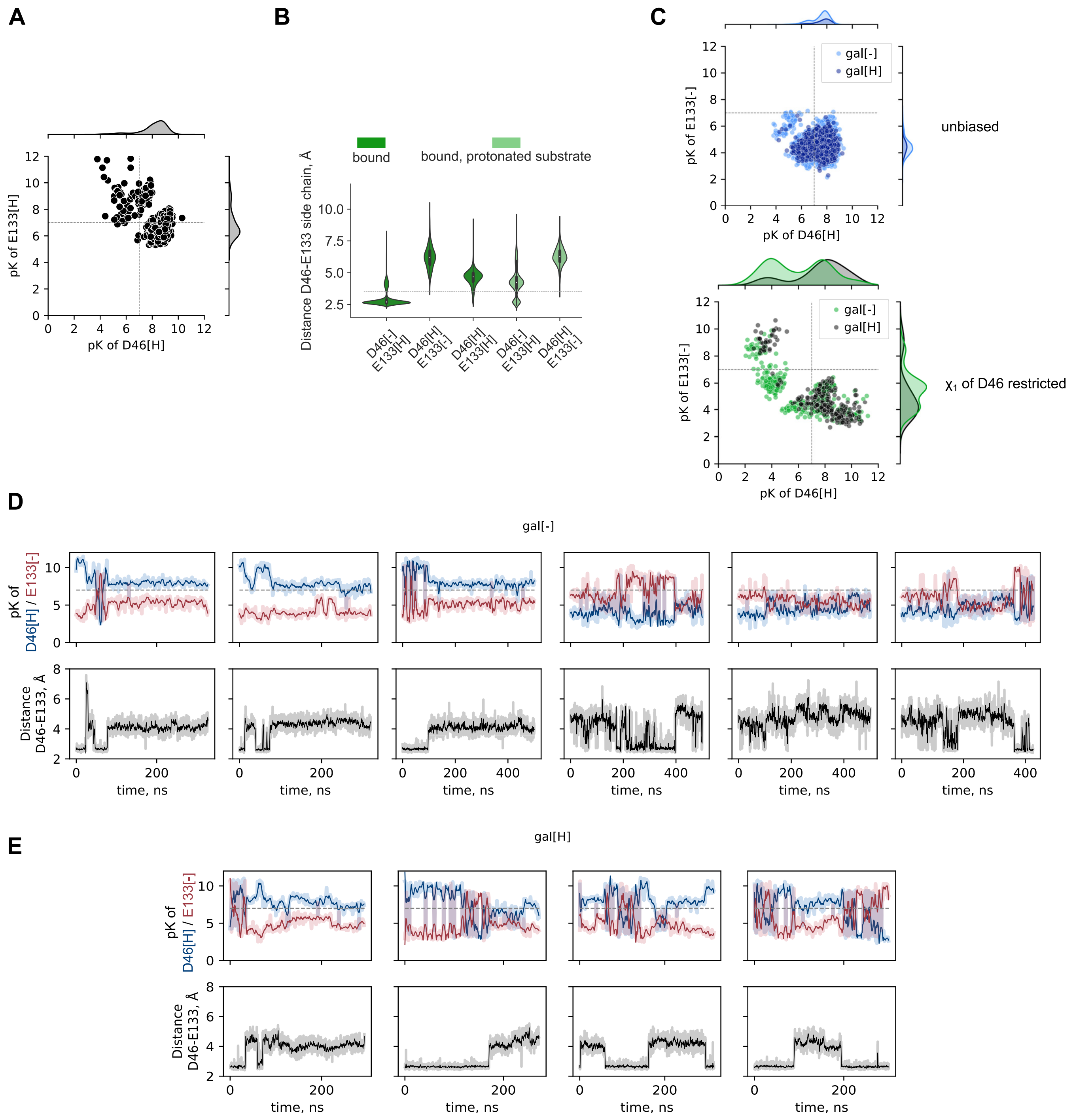

Figure EV4. pK_a calculations with the PROPKA tool.

(**A**) pK_a of E133 versus pK_a of D46 in simulations with inward-facing DgoT, D46 and E133 protonated and galactonate bound. (**B**) Probability densities for distances between side chains of D46 and E133 in simulations with inward-facing galactonate-bound DgoT (for simulations with deprotonated galactonate bound: $n = 10$ for simulations with only D46 or only E133 protonated, $n = 5$ for simulations with both D46 and E133 protonated; for simulations with protonated galactonate bound: $n = 4$). The central white dots represent the medians, thick black lines represent ranges between the 25th percentile (Q1) and the 75th percentile (Q3), the top and bottom points of the violin extend to the minimum and maximum values of the kernel density estimate (KDE). (**C**) pK_a of E133 versus pK_a of D46 in simulations with inward-facing DgoT with D46 protonated, E133 deprotonated and galactonate bound. *Top*: unbiased simulations, *bottom*: simulations with dihedral angle $\chi1$ of D46 restrained at $+70°$ (*close*-D46 conformation). (**D**, **E**) Time course of pK_a of D46 and E133 and distance between side chains of D46 and E133 in simulations with inward-facing DgoT with D46 protonated, E133 deprotonated and $\chi1$ of D46 restrained at $+70°$ (*close*-D46). Galactonate was present in the binding site in its deprotonated (**D**) or its protonated (**E**) form.

 © The Author(s)
